# Management of infection and ocular complications in pediatric SJS/TEN-like acute graft-versus-host disease: a clinical case study and literature review

**DOI:** 10.3389/fimmu.2025.1588297

**Published:** 2025-06-16

**Authors:** Huimin Yan, Yunjun Mo, Yue Li, Qian Li, Liping Luo, Qing Meng, Lei Jia, Lintao Zhou, Lixia Xiao, Xiaoying Fu

**Affiliations:** ^1^ Department of Laboratory Medicine, Shenzhen Children’s Hospital, Shenzhen, GuangDong, China; ^2^ Center of Clinical Laboratory, Shenzhen Hospital, Southern Medical University, Shenzhen, GuangDong, China; ^3^ Department of Pediatric Hematology & Oncology, Shenzhen Children’s Hospital, Shenzhen, GuangDong, China; ^4^ Center for Regenerative Medicine and Restorative Materials, Huangpu Institute of Materials, Guangzhou, China; ^5^ Guangxi Key Laboratory of Intelligent Precision Medicine, Nanning, China

**Keywords:** acute graft-versus-host disease, acute ocular graft-versus-host disease, fungi, infection, toxic epidermal necrolysis

## Abstract

Acute graft-versus-host disease (aGVHD) with skin manifestations reminiscent of Stevens-Johnson Syndrome (SJS) and Toxic Epidermal Necrolysis (TEN) is associated with poor outcomes. However, optimal management strategies to enhance quality of life in SJS/TEN-like aGVHD remain undefined. This study aims to investigate the management of complex infections and acute ocular injury in patients with SJS/TEN-like aGVHD following allogeneic hematopoietic stem cell transplantation. We conducted a comprehensive analysis of the treatment course for a patient with SJS/TEN-like aGVHD, complemented by a literature review on acute ocular complications and their management in aGVHD patients. A patient diagnosed with grade IV skin aGVHD received effective treatment for multidrug-resistant *Stenotrophomonas maltophilia* using minocycline, aztreonam, and ceftazidime-avibactam. Combination therapy with liposomal amphotericin B and voriconazole was efficacious against mixed fungal infections. Immunological assessments indicated reduced lymphocyte counts and increased myeloid-derived suppressor cells, with elevated CD4^+^ PD-1^+^ exhausted and memory cells, reflecting a complex interplay of immune hyperactivity and suppression. A literature review showed that although age, gender, and transplant circumstances were not associated with ocular symptoms, grade II+ cutaneous aGVHD emerged as a key risk factor for conjunctival involvement, characterized by exudation and pseudomembrane formation. Topical glucocorticoids, tacrolimus and cyclosporine eye drops were effective, necessitating regular pseudomembrane removal. Evaluating drug susceptibility and immune status is vital for formulating precise therapies. Early recognition and management of ocular symptoms in SJS/TEN-like aGVHD are essential to prevent irreversible damage.

## 1 Introduction

Graft-versus-host disease (GVHD) is an immune response characterized by donor-derived immune cells perceiving the recipient’s tissues as foreign, leading to immune-mediated damage. It remains one of the most severe complications of allogeneic hematopoietic stem cell transplantation (allo-HSCT), particularly acute graft-versus-host disease (aGVHD), which is associated with high morbidity and mortality (1). Classic aGVHD typically occurs within the first 100 days post-transplantation, although late-onset cases can also occur. It primarily affects the skin, gastrointestinal tract, and liver, with clinical manifestations including rash, abdominal pain, diarrhea, and elevated bilirubin levels (2). The severity of aGVHD is classified into four grades according to the Glucksberg grading system, with Grades 2–4 aGVHD having an overall incidence of 40-50%, and despite prophylactic measures, approximately 15% of patients develop severe, Grades 3–4 aGVHD (3). Opportunistic infections commonly occur after transplantation, such as those caused by herpes simplex virus and cytomegalovirus, or adverse drug reactions resulting from the use of antibiotics, nonsteroidal anti-inflammatory drugs (NSAIDs), immunomodulators, and targeted therapies, whose skin manifestations are often difficult to distinguish clinically from those of aGVHD (4, 5). Of particular concern are Stevens-Johnson Syndrome (SJS) and toxic epidermal necrolysis (TEN), which presents as a skin -mucosal reaction, characterized by painful erythema, bullae, and erosions that may extend to the limbs, while TEN can be accompanied by ocular symptoms including acute conjunctival hyperemia, erosion, and pseudomembrane formation (6). Several studies have reported that acute ocular graft-versus-host disease(oGVHD)can also present similarly, and given the differing management approaches for TEN and aGVHD, distinguishing between these two conditions is crucial (7–9). Previous large-scale studies based on SJS/TEN-like aGVHD have been significantly limited (8), and in this paper, we study the diagnosis of SJS/TEN-like aGVHD and the management of severe infection in a pediatric patient with mixed thalassemia approximately three months post cord blood-assisted haploidentical allo-HSCT, with regular monitoring of immune status to guide treatment. Furthermore, we provide a review and analysis of the literature concerning acute oGVHD, with the aim of contributing valuable insights for the treatment of severe aGVHD, which is still associated with high mortality rates, and for improving patient quality of life.

## 2 Case presentation

**Figure 1:**
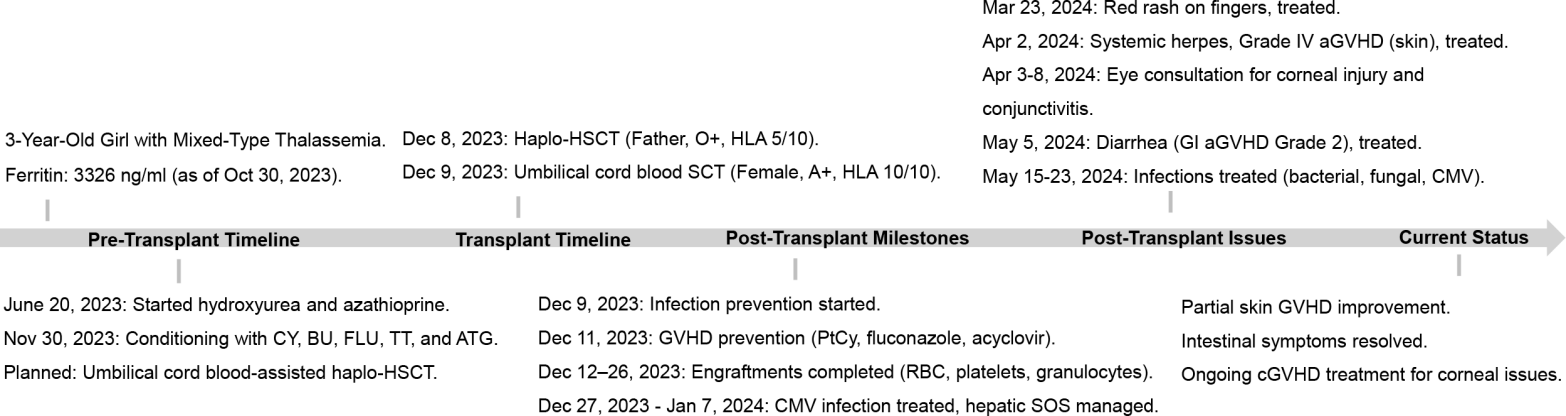
Patient transplantation status and disease progression. CY, cyclophosphamide; BU, busulfan; FLU, fludarabine; TT, thiotepa; ATG, anti-thymocyte globulin; PtCy, post-transplant cyclophosphamide; CNS, central nervous system; NGS, next-generation sequencing.

A 5-year-old female patient, initially diagnosed two years earlier with mixed-type thalassemia (-α3.7 deletion, β CD41-42/-28 mutation), had been receiving regular transfusion therapy. She was admitted to our hospital in preparation for hematopoietic stem cell transplantation(HSCT). Physical examination at admission revealed no abnormalities, while imaging demonstrated diffusely increased hepatic density and alterations in bone structure at the cranial base, paranasal sinuses, and thoracic bones, consistent with thalassemia. The progression of the diagnosis and treatment process is depicted in **Figure 1**.

## 3 Materials and methods

### 3.1 Lymphocyte transformation test

The modified lymphocyte transformation test (LTT) is an *in vitro* assay designed to evaluate drug sensitization in the context of patient allergy. Peripheral blood mononuclear cells (PBMCs) were freshly isolated from whole blood samples using Ficoll (LymphoPrep™) gradient centrifugation. Subsequently, 2 x 10^5^ PBMCs were cultured in 200 μL of RPMI-1640 (Gibco) medium supplemented with 10% fetal bovine serum, in a 96-well round-bottom plate. Trimethoprim-sulfamethoxazole was diluted in culture medium to obtain three concentrations: 21 μg/mL, 105 μg/mL, 210 μg/mL. Phytohemagglutinin (PHA) at 5 μg/mL was used as a positive control, while culture medium and DMSO (the solvent for the drug) served as negative controls. All conditions were set up in triplicate and incubated in a humidified incubator at 37°C with 5% CO^2^ for five days. On the fifth day, cell proliferation was assessed by counting viable cells using a TC20 Automated Cell Counter, cytokine concentrations in supernatants were analyzed by flow fluorescence luminescence and collected cells after centrifugation for subsequent experiments. The above drugs and DMSO were all obtained from the Sigma-Aldrich.

### 3.2 Antifungal susceptibility testing

*Candida parapsilosis*(*C. parapsilosis*) was determined using the Merieux AST-YS08 card. *Trichosporon asahii*(*T. asahii*) was determined using the Merieux ATB fungus3 card. *Fusarium solani* species complex(*FSSC*)was determined using E-test fungal susceptibility test strips.

### 3.3 Flow cytometry

Peripheral blood samples were collected using EDTA-2K as an anticoagulant to evaluate the patient’s immune regulation. The following antibodies were utilized: anti-human-CD45 V500, CD28-PE, CD4-PE-Cy7, CD3-APC, CD8-PerCP, CD38-FITC, CD45-V500, anti-human-CD3-APC-Cy7, PE-Cy7-CD27, CD4-V450, CD127-PE, CD25-APC, CD45RA-FITC, CD25-APC, and CD69-PE-Cy7 for immune evaluation. For spectral flow cytometry, the following antibodies were employed: anti-human-CD56-PE-CF594, CD16-BV786, CD14-APC, CD11b-PerCP-Cy5.5, HLA-DR-APC-Cy7, CD3-PE-Cy7, CD19-PE-Cy5, CD4-FITC, CD8-BV711, CD45RA-BV510, CCR7-AF700, CD38-PE, and PD-1 PE-Fire640 for surface staining. Antibodies and specimens were thoroughly mixed and incubated at room temperature for 15 minutes, protected from light. Subsequently, 1 mL of lysis buffer was added for red blood cell removal, followed by a 15-minute incubation away from light. The samples were washed twice with 2 mL of PBS and centrifuged at 1500 rpm for 5 minutes. After removing the supernatant, 300 μL of PBS was added in preparation for testing. Analysis was performed using a FACS Canto II cytometer (BD Biosciences, San Jose, CA, USA). Spectral flow cytometry data were collected on a Cytek Aurora/NL (Cytek Biosciences) using Cytek SpectroFlo software. All antibodies were obtained from Becton Dickinson (Mountain View, CA, USA) and Beckman Coulter (Brea, CA, USA), and data analysis was conducted using FlowJo 10.0.

### 3.4 Literature review of acute oGVHD

We conducted a literature review on aGVHD with ocular symptoms using PubMed (http://www.ncbi.nlm.nih.gov) up to March 2025. The search term applied was “(Acute Ocular Graft-versus-Host Disease) OR (Conjunctival Acute Graft-versus-Host Disease).” Only studies involving patients diagnosed with aGVHD presenting with acute ocular symptoms were included in the analysis. We summarized patient characteristics, types of transplantation, pre-transplantation conditioning, and prevention measures. Our focus was on collecting data on acute ocular manifestations and treatment outcomes to support early clinical examination of ocular involvement in aGVHD, enabling timely intervention to enhance post-transplant quality of life.

## 4 Results

### 4.1 Diagnosis of grade IV aGVHD

**Figure 2:**
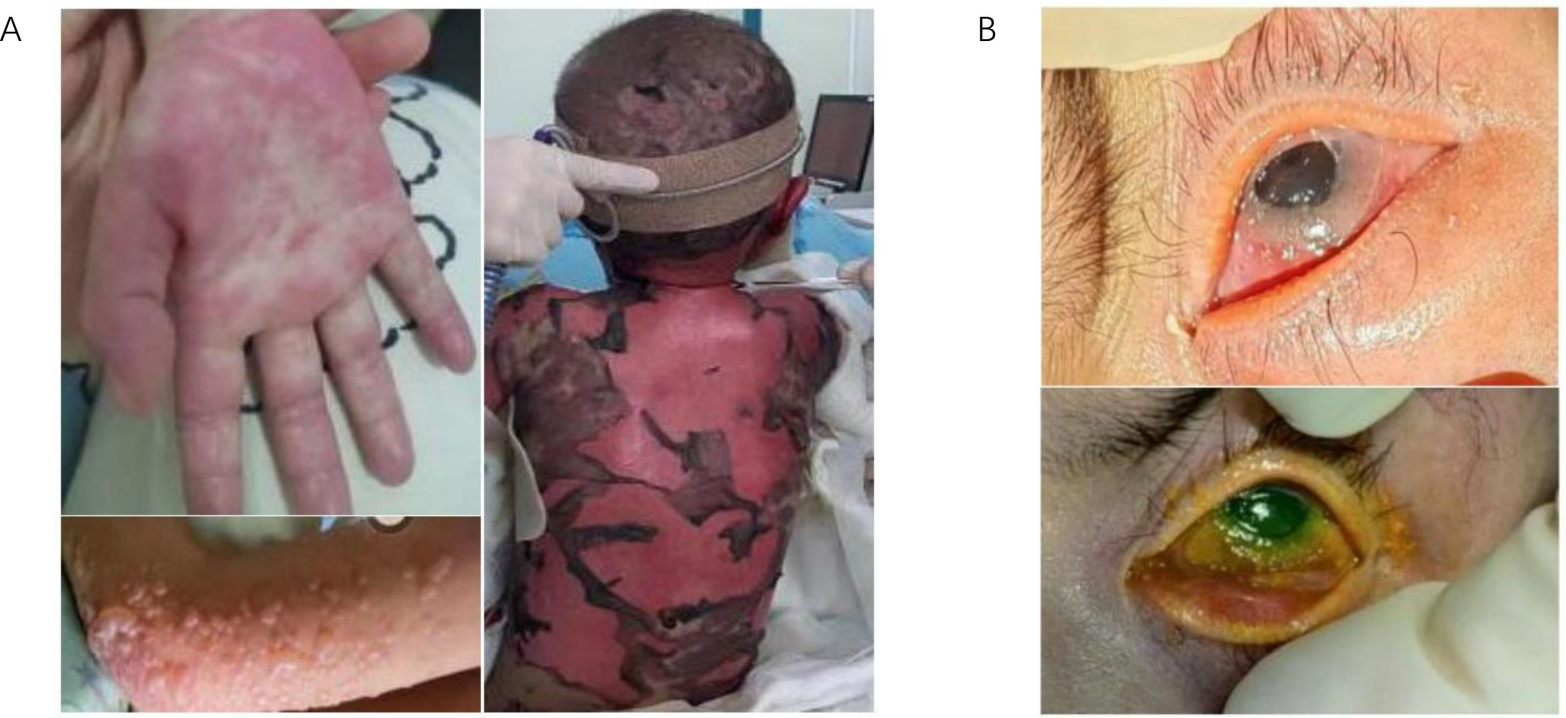
Manifestations of skin and ocular symptoms in the patient with SJS/TEN-like aGVHD. **(A)** The skin shows a sequential progression of symptoms, starting with red rashes, followed by the appearance of blisters, and ultimately extensive necrosis and desquamation. **(B)** the patient’s eyes presented with corneal damage and pseudomembrane formation.

The patient’s skin rash manifested as bullous lesions followed by ulceration, consistent with SJS or TEN (**Figure 2**). The characteristic features included mucositis, widespread targetoid erythema, bullae formation, and epidermal detachment. At this stage, cytokine levels were measured in both blister fluid and blood samples, which revealed elevated levels of inflammatory cytokines (**Supplementary Table S1**). The Patient carrying candidate HLA genes for severe skin reactions induced by cotrimoxazole after transplantation. To investigate whether the rash was triggered by drug hypersensitivity, an activation test using peripheral blood mononuclear cells was performed; however, the results did not support this hypothesis (**Supplementary Figure S1**, **Supplementary Tables S2**, **S3**). According to the modified Glucksberg grading system for aGVHD, hepatic involvement was absent, and gastrointestinal symptoms were mild, corresponding to grade II involvement, whereas the skin manifestations were consistent with grade IV aGVHD. Consequently, the overall diagnosis was established as grade IV aGVHD. The patient also developed acute immune-mediated ocular complications, characterized by conjunctival hyperemia and extensive damage with exfoliation of the lower corneal region, accompanied by increased eyelid secretions (**Figure 2**).

### 4.2 Serious bacterial and fungal infections

**Figure 3:**
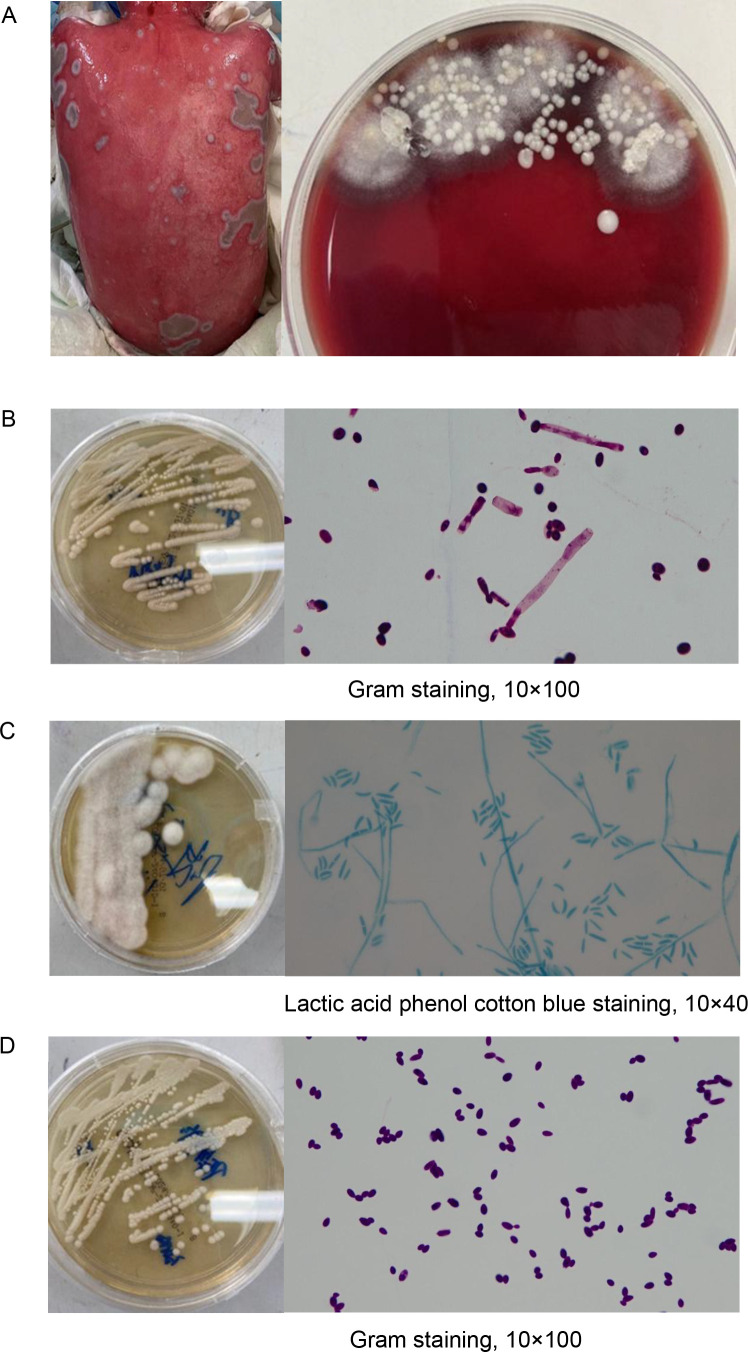
Identification of multiple fungal species in skin exudate culture. **(A)** Shown are the morphology of skin secretions cultured in blood plates until day 4, and then individual colonies were isolated onto Sabouraud Dextrose Agar and identified by matrix-assisted laser desorption/ionization time-of-flight mass spectrometry (MALDI-TOF MS), respectively. **(B)** *Trichosporonas asahii. (*C*) Fusarium solani* species complex. **(D)** *Candida parapsilosis*.

One month following the onset of aGVHD, the patient’s skin exudate culture revealed the presence of *S. maltophilia*. Despite treatment with a combination of minocycline and cefoperazone-sulbactam sodium, the patient continued to exhibit elevated inflammatory markers and core body temperature. Based on the antimicrobial susceptibility testing results (**Supplementary Figure S2**, **Supplementary Table S4**), cefoperazone-sulbactam sodium was substituted with a combination regimen comprising aztreonam and ceftazidime-avibactam, alongside continued administration of minocycline, to target resistant *S. maltophilia*. By the second day of treatment, the patient’s core body temperature had normalized, and inflammatory markers had significantly declined. Subsequently, large clusters of white plaques appeared on the skin of the patient’s chest, neck, and back. Microbiological cultures identified three fungal species: *C. parapsilosis*, *FSSC*, and *T. asahii* (**Figure 3**). Notably, the first two fungi were also detected in stool samples. Following a review of relevant literature and antifungal susceptibility testing, caspofungin was discontinued, and liposomal amphotericin B combined with voriconazole was initiated for antifungal therapy. After one month of targeted antifungal treatment, fungal cultures from the patient’s skin were negative, with normal commensal bacteria observed in their place. However, subsequent blood cultures revealed *C. parapsilosis* infection, which was successfully treated, converting to negative after an additional month of therapy. Despite the occurrence of severe skin and bloodstream infections, the precise and timely results of antimicrobial susceptibility testing effectively guided the therapeutic interventions for this patient (**Supplementary Table S5**).

### 4.3 Immune monitoring and treatment

**Figure 4:**
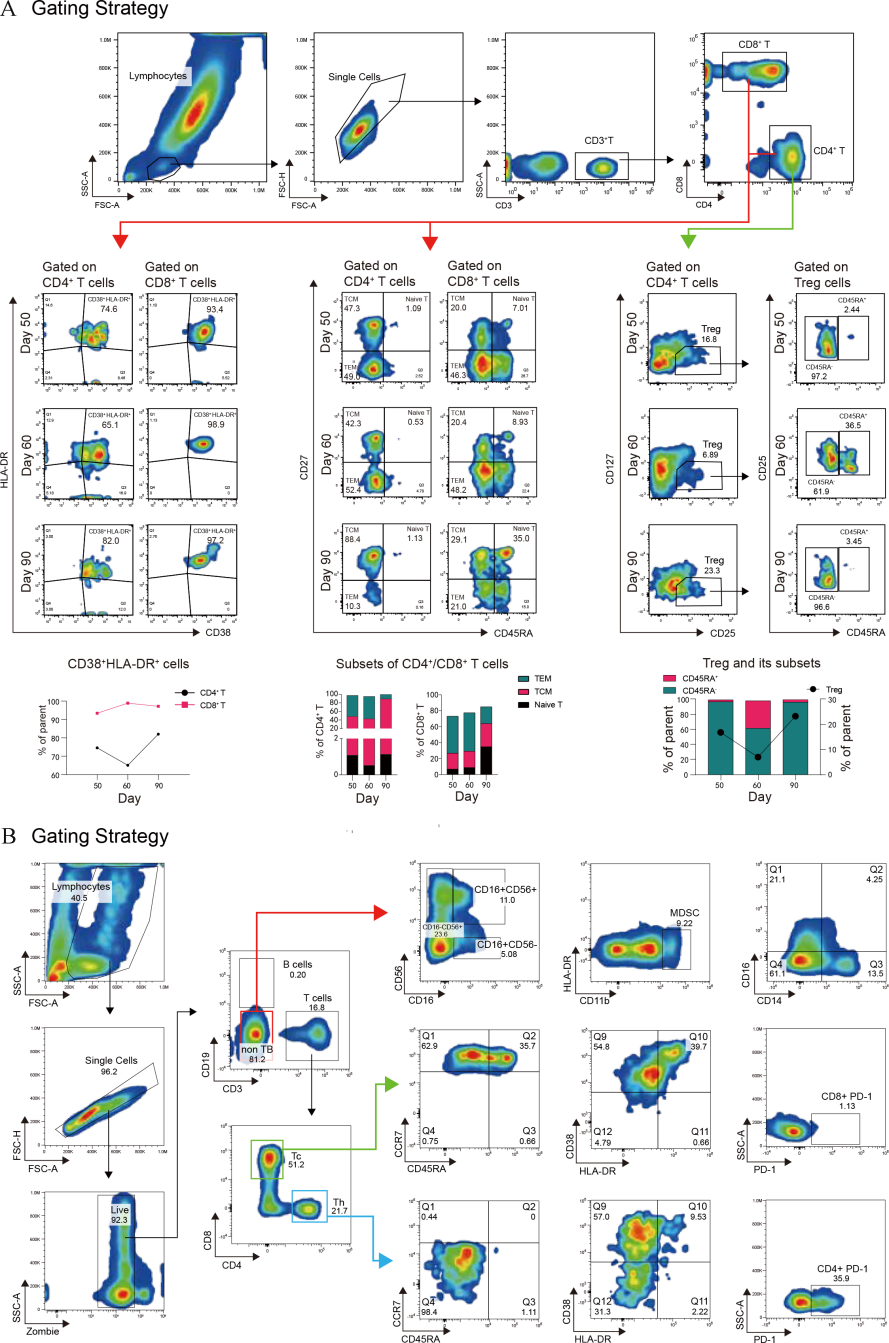
Immune system monitoring following aGVHD onset. **(A)** Activation cells were identified by the co-expression of CD38 and HLA-DR. Trends in activation molecule expression at different time points are illustrated with a line graph and data points. Naive T cells (CD45RA^+^CD27^+^), central memory T cells (TCM, CD45RA^-^CD27^+^), and effector memory T cells (TEM, CD45RA^-^CD27^-^) were analyzed using flow cytometry, with results presented as histograms. **(B)** Spectral flow cytometry was employed to evaluate both innate and adaptive immune cell subsets, as shown by the gating strategy in the figure. This analysis includes NK cell subsets, monocyte subsets, myeloid-derived suppressor cells (MDSCs), CD4^+^ and CD8^+^ naive, memory, and activated T cells, as well as the expression of the exhaustion marker PD-1 on CD4^+^ and CD8^+^ T cells.

During the course of treatment, we monitored the patient’s immune status using flow cytometry, revealing a persistent state of concurrent immune activation and suppression. As illustrated in **Figure 4**, following the onset of aGVHD, the patient’s T cell activation state persisted, with a continual increase in the proportion of CD8^+^ CD38^+^ HLA-DR^+^ T cells. The distribution and proportion of CD4^+^ and CD8^+^ T cell subsets underwent significant changes over time on Day 50, 60, and 90, with both CD4^+^ and CD8^+^ T cell subsets displaying similar trends. Notably, central memory T cells increased followed treatment, while the percentage of CD4^+^ effector memory T cells peaked on Day 60 and subsequently declined by Day 90, and treatment with tacrolimus led to an increase in regulatory T cells (Tregs) (**Supplementary Table S6**), as expected. Spectral flow cytometry analysis of the patient’s immune system on day 90 showed a reduction in lymphocyte proportion, an increase in myeloid-derived suppressor cells (MDSCs), and elevated proportions of CD4^+^ PD-1^+^ exhausted cells and memory cells, indicating an immune dysregulation state characterized by simultaneous immune hyperactivity and suppression. Following aggressive treatment, partial remission was observed in skin immune rejection, complete remission in gastrointestinal symptoms, whereas the acute ocular manifestations, specifically corneal damage, progressed and worsened due to delayed intervention, leading to functional loss. Ultimately, the patient entered the chronic graft-versus-host disease (cGVHD) treatment phase, with the transplantation status and associated treatment outlined in **Figure 1**.

### 4.4 A review of literature on acute oGVHD

Acute oGVHD represents a rare complication of aGVHD, occurring in approximately 7.2% of allo-HSCT recipients (10). In our systematic review, a predefined keyword-based search strategy in PubMed initially retrieved 132 potentially relevant articles. After screening excluding studies with inadequate clinical documentation of acute oGVHD and non-SCI-indexed publications, only 10 articles met our inclusion criteria, including merely 6 case reports providing patient-level evidence (**Table 1**). The analysis included data from 59 transplant recipients, 28 of whom had documented aGVHD grading. Of these, 22 patients were classified as grade II-IV. Notably, previous studies have identified grade II or higher skin aGVHD as a significant risk factor for the development of conjunctival aGVHD (11). Although retrospective analyses revealed no significant associations between ocular symptom onset and patient demographics (age/sex), transplant characteristics, or conditioning regimens, case reports also suggested no apparent link to CMV infection. Acute oGVHD typically develops subsequent to systemic aGVHD onset, demonstrating characteristic clinical manifestations including ocular pain, epiphora, conjunctival hyperemia, pathological ocular surface exudation, corneal ulceration, and pseudomembrane formation - features that are clinically distinct from cGVHD presentations. The therapeutic regimen comprised combined topical and systemic corticosteroid administration augmented by ocular tacrolimus application. Scheduled pseudomembrane debridement was implemented to mitigate scarring risk, complemented by adjunctive topical cyclosporine therapy. Symptomatic management included artificial tear instillation and amniotic membrane lens application. The patient described in this report exhibited grade IV cutaneous aGVHD with concurrent severe ocular involvement, manifesting as marked conjunctival hyperemia, significant corneal epithelial damage, and profuse inflammatory ocular discharge.

**Table 1:** Characteristics of aGVHD with ocular symptoms in patients undergoing HSCT.

No.	Year	Ref Type	Pt Details	Underlying Cond.	Tx Type	Pre-Tx Cond.	GVHD Prophylaxis	aGVHD Grading	Onset of aGVHD	Ocular Sx	CMV Inf	Tx	Outcome
1	1996 (41)	Case	44y, M	CML	Allo-BMT	120 mg/kg Cy, 10 Gy single	MTX, CsA	Gr III, day 40	Day 45	Eye burning, blurred vision, conjunctival lesion	No	Topical steroids + systemic therapy	Deceased on day 58 (bloody diarrhea, diffuse skin erythema)
2	2002 (45)	Case	26y, F	AML M2	MRD	Cy, Bu, TBI	MTX, CsA	Gr II, day 18	Day 38	Eye pain, lacrimation, corneal ulcers, pseudomembranes	No	FK506, DLI	Deceased on day 202
3	2002 (46)	Case	30y, M	HL	ASCT			Gr I, day 42	Day 63	Conjunctival injection, corneal epithelial defects	No	FK506, systemic steroids, topical CsA, artificial tears	CR
4	2016 (11)	Res	9/F,4/M	ALL, AML, DLBCL, MM	MS n=5, UMS n=8	RIC n=5, MAC n=8		Gr I (n=2), II (n=1), III (n=7), IV (n=3)	Day 20-142	Stage 1–2 (n=5), Stage 3–4 (n=8)	3 (–), 10 (+)		8 alive, 5 deceased
5	2017 (47)	Case	5y, M	Stage IV NB	Haplo-HSCT	TBI, Cy	Tacrolimus, MTX (short-term)	Gr II, day 28	Day 26	Eyelid swelling, ocular pruritus, conjunctival hyperemia	No	Topical steroids, systemic prednisolone, tacrolimus	Improvement within 5 weeks
6	2017 (42)	Case	21y, M	AML	Haplo donor			Gr IV, day 21	Day 30	Pseudomembranes in tarsal conjunctivae	No	Pseudomembrane removal, glucocorticoids, additional immunosuppression	Symptoms alleviated, chronic GVHD developed
7	2018 (43)	Res	8/F,13/M	ALL, AML, MDS, CML, HL, AA	Haplo-HSCT	TBI, Cy	Tacrolimus, MTX (short-term)		< 2 months	Increased mucus secretion, bulbar conjunctival congestion, lacrimation	No		
8	2022 (48)	Res	2/F, 7/M	ALL, AML, MDS, PID	Related donor (n=3), Unrelated donor (n=6)	Bu, TBI, ATG, Flu		Gr I (n=3), II (n=1), III (n=2), IV (n=3)	Day 8-122 (median 38)	Stage 1 (n=3), Stage 2 (n=3), Stage 4 (n=3)			2 developed chronic oGVHD
9	2022 (44)	Res	4/F, 6/M							Abrupt eye redness, purulent exudate, eye pain, false membranes			
10	2025 (49)	Case	11y, M	ALL	unrelated donor	60 mg/kg Cy, 12 Gy	MTX, CsA	Gr III	Day 27	Conjunctival injection, blurred vision		Systemic prednisolone, IVIg, cyclosporine 0.05% eye drops, amniotic membrane lenses	Approximately 6 months, CR

Ref Type, Reference Type; Pt Details, Patient Details; Underlying Cond., Underlying Condition; Tx Type, Transplant Type; Pre-Tx Cond., Pre-transplant Conditioning; aGVHD, acute Graft-versus-Host Disease; Ocular Sx, Ocular Symptoms; CMV Inf, Cytomegalovirus Infection; Tx, Treatment; Case Rep, Case Report; Res, Research; CML, Chronic Myelogenous Leukemia; MRD, Matched Related Donor; Cy, Cyclophosphamide; Bu, Busulfan; TBI, Total Body Irradiation; MTX, Methotrexate; CsA, Cyclosporine; FK506, Tacrolimus; IVIg, intravenous immunoglobulin; DLI, Donor Lymphocyte Infusion; CR, Complete Response; MAC, Myeloablative Conditioning; RIC, Reduced-intensity Conditioning; MS, Matched sibling; UMS, Unmatched sibling; NB, Neuroblastoma; HSCT, Hematopoietic Stem Cell Transplant; ASCT, autologous stem cell transplantation; AML, Acute Myeloid Leukemia; ALL, Acute Lymphoblastic Leukemia; DLBCL, Diffuse Large B-Cell Lymphoma; MM, Multiple Myeloma; MDS, Myelodysplastic Syndrome; PID, Primary Immunodeficiency; ATG, Anti-Thymocyte Globulin; Flu, Fludarabine.

## 5 Discussion

The patient developed a generalized rash that progressed to necrosis and sloughing, resembling TEN. Both TEN and TEN-like manifestations of GVHD are rare but life-threatening complications in patients undergoing allo-HSCT (8). These two conditions share similar pathophysiological mechanisms primarily involving the activation of cytotoxic T cells, leading to extensive cytokine and cytotoxic protein infiltration, which in turn results in skin necrosis (12). Clinically, both conditions present with highly similar symptoms and histopathological findings upon skin biopsy. TEN typically occurs between 4 to 28 days following drug exposure, while aGVHD manifests approximately 3 to 5 weeks post-transplantation. This patient excluded autoimmune skin diseases and infectious factors causing TEN (including *Mycoplasma pneumoniae* and herpes simplex virus) (13). Chimerism analysis revealed the patient is in a complete donor chimerism state with 70% paternal and 30% umbilical cord blood stem cells, and HLA typing showed the presence of donor-derived HLA-A*11:01 and HLA-C*06:02, which have previously been associated with severe adverse cutaneous reactions including the co-trimoxazole-induced SJS/TEN (14, 15). The patient had been receiving trimethoprim-sulfamethoxazole for over three months as prophylaxis against opportunistic infections such as *Pneumocystis jirovecii* pneumonia following transplantation. Given the potential for an adverse drug reaction, causality assessment using the ALDEN score and Naranjo algorithm indicated a “possible” association between trimethoprim-sulfamethoxazole and SJS/TEN (16, 17). However, during treatment, the modified lymphocyte transformation test was negative, suggesting that trimethoprim-sulfamethoxazole is less likely to be the causative agent of TEN, though clinical correlation remains essential given the limitations of LTT. The disease initially presented with erythema that began on the fingers and progressively extended to the ears, elbows, knees, ankles, and soles, while the distal limbs of SJS/TEN were relatively unaffected. Pancytopenia(which may result from drug-induced myelotoxicity or viral infections in transplant recipients), pneumonia, and gastrointestinal reactions that emerged one month after the initiation of treatment. These clinical manifestations usually appear in patients with SJS/TEN-like aGVHD rather than SJS/TEN (8). Despite the patient’s HLA alleles being linked to sulfonamide hypersensitivity, no allergic reaction occurred, potentially due to concurrent administration of immunosuppressants such as methylprednisolone and tacrolimus, which are commonly used in the management of drug-induced hypersensitivity syndrome. This suggests that immune tolerance may have developed due to multiple exposures in the context of immunosuppression. In 2018, Maira Fonseca et al. conducted a seven-year study involving 831 patients undergoing allo-HSCT (18), and found that expression of HLA-A*0101 by the donor or recipient was associated with increased incidence and severity of cutaneous aGVHD, particularly grade III-IV skin involvement. Similarly, in our case, the donor expression of HLA-A*0101, suggesting a possible link to the patient’s severe, refractory cutaneous aGVHD. Although the precise mechanisms by which HLA gene polymorphisms influence cutaneous aGVHD remain unclear, this finding has potential implications for developing preventive or early intervention strategies for cutaneous aGVHD in high-risk individuals, warranting further investigation. Timely and accurate diagnosis of TEN in transplant patients is crucial. To address this challenge, a comprehensive analysis is required, including a thorough physical examination of the rash, skin biopsy, colonoscopy, review of drug history, application of drug causality algorithms, lymphocyte activation tests, viral serological tests, and chimerism studies.

The patient exhibited multiple risk factors, including high-dose corticosteroid therapy, CMV infection or reactivation, Grade II or higher aGVHD, prolonged neutropenia, and umbilical cord blood transplantation. These factors collectively heightened the risk of immune checkpoint inhibitor infections, in accordance with findings reported in a recent meta-analysis (19). *S. maltophilia* is a gram-negative opportunistic pathogen with intrinsic resistance to multiple antibiotics, including cephalosporins and carbapenems. Reports of soft tissue infections caused by *S. maltophilia* in immunocompromised patients are rare (20), and treatment of moderate to severe infections generally requires combination therapy. A multicenter study found that the longest survival times in children with severe infections due to *S. maltophilia* were associated with a treatment regimen combining ciprofloxacin, trimethoprim-sulfamethoxazole, and minocycline. Aztreonam-avibactam (21), currently in Phase II clinical development, is considered a promising alternative therapy for *S. maltophilia* associated infections (22). Several studies suggest that the combination of aztreonam with ceftazidime-avibactam provides a novel treatment approach for resistant strains (23–25). In cases of multidrug-resistant (MDR) *S. maltophilia* with limited therapeutic options, combination therapy with aztreonam and ceftazidime-avibactam should be considered (24). The most common *Fusarium* species is *FSSC* (26), which frequently invades immunocompromised individuals, such as patients with hematological malignancies, and has high mortality rates. In recent years, *Fusarium* species has demonstrated reduced susceptibility to existing antifungal agents. Voriconazole has shown the highest *in vitro* antifungal activity, followed by amphotericin B (27). According to the Clinical and Laboratory Standards Institute (CLSI) epidemiological cutoff values described by Espinel-Ingroff et al (28), the minimum inhibitory concentrations (MICs) of this strain for voriconazole and amphotericin B are 1 μg/mL and 2 μg/mL, respectively, were well below the established cutoff values. *T. asahii*, a yeast-like fungus, is rarely associated with superficial skin infections in children compared to systemic infections (29). Previous case reports have shown successful treatment of *T. asahii* infections using voriconazole monotherapy, making it the preferred treatment option (29). Amphotericin B may also be used in combination (30). According to the CLSI guidelines, *in vitro* susceptibility testing of the isolated strain showed that both voriconazole and amphotericin B were effective, consistent with antifungal susceptibility characteristics for Trichosporon species in China (31). Despite the patient receiving prophylactic treatment with caspofungin, both fungi were identified as inherently resistant to this agent. Studies have demonstrated that *C. parapsilosis* is the most common species responsible for breakthrough invasive candidiasis in patients treated with echinocandins (32). Colonization of the gastrointestinal tract or skin is the first step, and echinocandin therapy may promote *C. parapsilosis* growth by reducing the competing microbiota, thereby increasing the likelihood of breakthrough infections. Candidemia due to reduced echinocandin susceptibility of *C. parapsilosis* has also been reported (32). Here, we report, for the first time, the simultaneous detection of three different fungi on the skin of a patient with TEN-like aGVHD. After one month of treatment with voriconazole in combination with liposomal amphotericin B, repeated fungal cultures of skin secretions were negative. Unfortunately, the patient developed an endogenous infection due to several risk factors, with *C. parapsilosis* detected in the bloodstream, necessitating continued antifungal treatment. The successful treatment of this complex infection emphasizes the necessity of strict management of antimicrobial use and monitoring of antimicrobial susceptibility.

The patient received GVHD prophylaxis with post-transplant cyclophosphamide (PtCy), tacrolimus, and mycophenolate mofetil, a regimen that previous studies have shown can effectively reduce the incidence of GVHD (33). In this case, the prophylaxis failed, suggesting that successful combinations may depend on optimizing the PtCy dose (34). Recent research focuses on combining PtCy-based strategies with novel agents, such as incorporating vedolizumab or abatacept into low-dose PtCy regimens (35). Additionally, the selective JAK1 inhibitor Itacitinib has been combined with PtCy/TAC/MMF for haploidentical transplantation, and none of the recipients experienced grade 3–4 aGVHD. First-line treatment for aGVHD is glucocorticoids, and recently, combination therapies for frontline treatment have received expert recommendations (36). For instance, glucocorticoids combined with the JAK2 inhibitor ruxolitinib not only demonstrate high response rates but also reduce steroid exposure, mitigate toxicity, and improve long-term survival. In this patient, rashes appeared on the extremities, prompting the addition of ruxolitinib alongside glucocorticoids; however, the symptoms did not improve, and the rash and subsequent exudation worsened. This lack of response may be related to the concomitant use of the antifungal agent voriconazole, which interferes with ruxolitinib metabolism (37)—a hypothesis requiring further investigation. Previous studies indicate that the primary adverse effects of ruxolitinib include thrombocytopenia, anemia, and neutropenia (38). The patient was refractory to glucocorticoid treatment, resulting in steroid-refractory (SR) aGVHD. Studies report that even with the use of ruxolitinib, approved in 2019 for SR-aGVHD (39), prognosis remains poor, with a median survival time of 11 months. Following the onset of GVHD, the patient’s immune status underwent significant alterations, including a decline in the proportion of lymphocytes, which likely reflects the disease’s immunosuppressive effect. Meanwhile, there was an increase in MDSCs, which are known to suppress T cell responses and facilitate immune evasion by tumors, potentially exacerbating GVHD in the inflammatory state. Innate immune components, such as NK cells and monocyte subpopulations, may also be altered during GVHD, potentially impacting early immune responses and contributing to overall immune dysregulation. Analysis of T cell subsets revealed increased levels of CD4^+^ and CD8^+^ T cells expressing the exhaustion marker PD-1, suggesting a state of T cell exhaustion characterized by diminished functionality following chronic antigen exposure, which impairs the ability to control GVHD. Conversely, there was an increase in the proportion of memory T cells, which are critical for long-term immune protection, suggesting an adaptive immune response to the persistent antigen stimulation induced by GVHD. Immunosuppressants, such as tacrolimus, can modulate this immune response, leading to an increase in Tregs, which are essential for maintaining immune homeostasis and mitigating excessive immune responses. The increase in Tregs, particularly in the induced subset, could be attributed to tacrolimus’s therapeutic action, aimed at controlling hyperactive immune responses while preventing immunosuppression. In conclusion, encompasses a multifaceted and intricate interplay among immune cells and their respective functions. A profound comprehension of the alterations within immune subpopulations is essential for devising targeted therapeutic interventions. These interventions are designed to recalibrate the immune equilibrium, and consequently, enhance clinical outcomes for patients.

Our approach involved cyclophosphamide and anti-thymocyte globulin (ATG) to deplete T cells and prevent rejection, along with decitabine (DAC) treatment. Beyond its antitumor properties, DAC has demonstrated potential immunomodulatory effects, with studies suggesting that low-dose DAC can reduce T cell proliferation and pro-inflammatory cytokine production, potentially ameliorating GVHD by enhancing immune tolerance (40). Simultaneously, we actively managed infections and immunosuppression to achieve equilibrium. For severe inflammation caused by GVHD, we employed tocilizumab to antagonize the IL-6 receptor, thereby suppressing inflammation, while recombinant anti-CD25 antibodies were used to bind IL-2 receptor alpha and inhibit T lymphocyte activation, exerting an anti-GVHD effect. In response to secondary gastrointestinal involvement, we mainly utilized basiliximab in combination with glucocorticoids and tacrolimus, leading to partial symptom relief. Overall, a comprehensive understanding of the immune mechanisms underlying GVHD and the effective management of its complications are paramount to improving outcomes for affected patients. The therapeutic approach we adopted highlights the need for ongoing innovation in the combination of immunosuppressive agents and targeted therapies to control GVHD while ensuring the maintenance of immune equilibrium.

During the acute phase, patients exhibited conjunctival hyperemia and pseudomembrane formation due to the consolidation of exudate over ulcerated necrotic surfaces. Initially, these symptoms complicated the differential diagnosis between aGVHD and TEN (41), as both conditions share similar clinical manifestations. Despite regular ophthalmologic evaluations every 2–3 days and concurrent treatment with topical ofloxacin eye drops and erythromycin ointment, the potential diagnosis of acute immune-mediated ocular rejection was not established until more than 30 days after symptom onset. Treatment was then supplemented with eye drops containing tacrolimus and betamethasone for eyelid care. Previous reports have documented successful treatment in adults diagnosed with Grade 4 aGVHD (42), in which conjunctival pseudomembranes were present on both the upper and lower tarsal plates, and local prednisolone therapy, along with periodic pseudomembrane debridement, led to complete resolution after three weeks. In the present case, oGVHD was diagnosed and treated late. One of the contributing factors to this delay was the prioritization of managing life-threatening, complicated infections. A literature review revealed that patients with ocular aGVHD often present with ocular erythema and increased discharge (42–44), frequently resulting in misdiagnosis as simple conjunctivitis, leading to subsequent treatment with antibiotics. Currently, there is no standardized diagnostic criterion for ocular aGVHD, primarily due to the rarity of studies on this condition. Its incidence is significantly lower compared to ocular cGVHD, and subjective clinical judgment by ophthalmologists often results in delayed treatment and diagnostic challenges. Consequently, clinicians must recognize that aGVHD can involve ocular structures in addition to its classic cutaneous, hepatic, and gastrointestinal manifestations, necessitating a multidisciplinary team approach for optimal management of patients with SJS/TEN-like presentations.

In summary, patients with aGVHD exhibiting SJS/TEN-like features are particularly vulnerable to life-threatening infections due to extensive skin exfoliation, the consequent loss of the primary defense barrier, and the use of immunosuppressive therapies, which further increase susceptibility to fungal and viral pathogens. Prompt and precise treatment is essential for a favorable outcome. Furthermore, the higher the severity of aGVHD, especially with skin involvement, the more critical it becomes to pay attention to acute ocular manifestations, particularly in pediatric patients. As children often have a longer expected post-transplant lifespan, addressing ocular complications in this age group is paramount. Early identification and treatment of ocular aGVHD are vital to prevent permanent tissue damage, which is essential for maintaining quality of life in the long term.

## Data Availability

The original contributions presented in the study are included in the article/**Supplementary Material**. Further inquiries can be directed to the corresponding author.
