# Supplementary material for: Management of infection and ocular complications in pediatric SJS/TEN-like acute graft-versus-host disease: a clinical case study and literature review
Source: Front Immunol. 2025 Jun 16;16:1588297. doi: 10.3389/fimmu.2025.1588297 (PMC12206640; doi:10.3389/fimmu.2025.1588297)
Supplement: Supplementary file 1 [file DataSheet1.docx]

Management of Infection and Ocular Complications in Pediatric SJS/TEN-like Acute Graft-versus-Host Disease: A Clinical Case Study and Literature Review

Huimin Yan, Yunjun Mo, Yue Li, Qian Li, Liping Luo, Qing Meng, Lei Jia,Lintao Zhou,Lixia Xiao,Xiaoying Fu

**Materials and experimental methods**

The modified lymphocyte transformation test (LTT) is an in vitro assay designed to evaluate drug sensitization in the context of patient allergy. Peripheral blood mononuclear cells (PBMCs) were freshly isolated from whole blood samples using Ficoll (LymphoPrep™) gradient centrifugation. Subsequently, 2 x 10⁵ PBMCs were cultured in 200 μL of RPMI-1640 medium supplemented with 10% fetal bovine serum, in a 96-well round-bottom plate. Trimethoprim-sulfamethoxazole was diluted in culture medium to obtain three concentrations: 21 μg/mL, 105 μg/mL, 210 μg/mL. Phytohemagglutinin (PHA) at 5 μg/mL was used as a positive control, while culture medium and DMSO (the solvent for the drug) served as negative controls. All conditions were set up in triplicate and incubated in a humidified incubator at 37°C with 5% CO₂ for five days. On the fifth day, cell proliferation was assessed by counting viable cells using a TC20 Automated Cell Counter, and flow cytometry was employed to analyze both the supernatant and the cells collected after centrifugation.


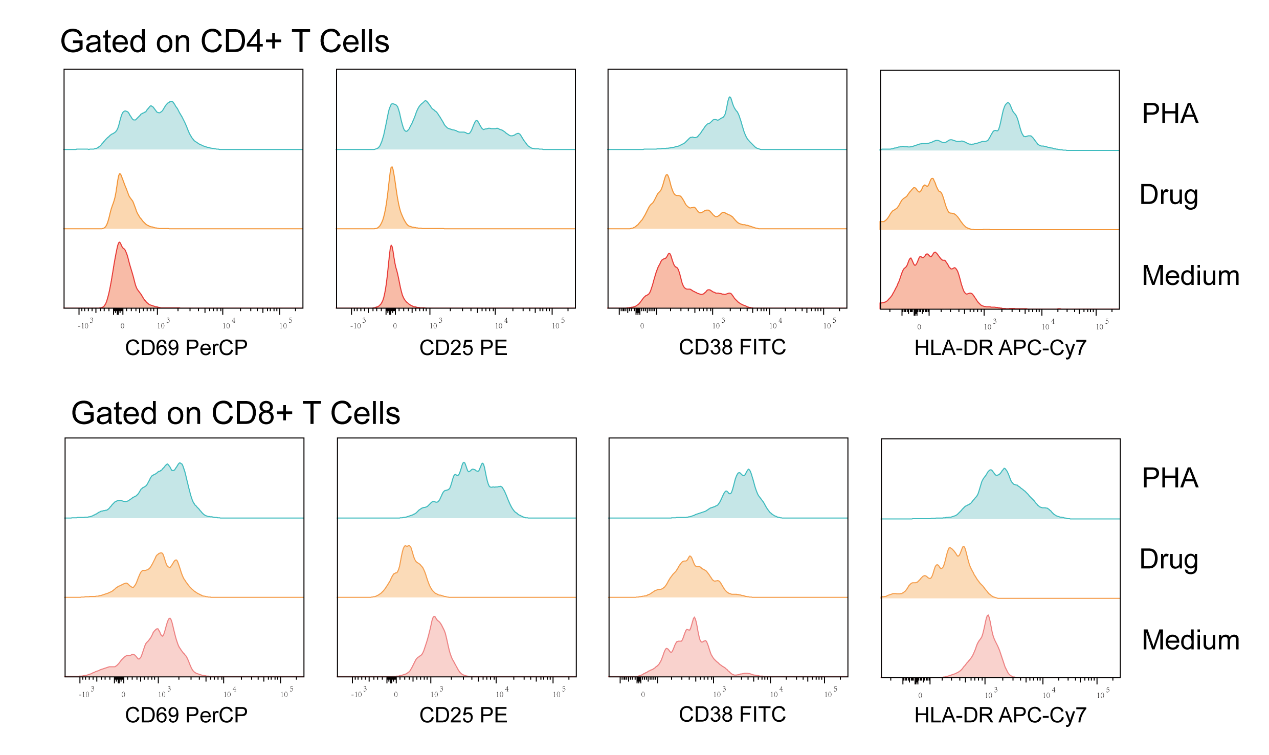


**Supplementary figure 1** Effects of Phytohemagglutinin (PHA) and drug stimulation on activation markers in CD4⁺and CD8⁺ T Cells. Expression of CD69, CD25, CD38, and HLA-DR demonstrates no up-regulation induced by the drug(The concentration of trimethoprim-sulfamethoxazole was 105 μg/ml).


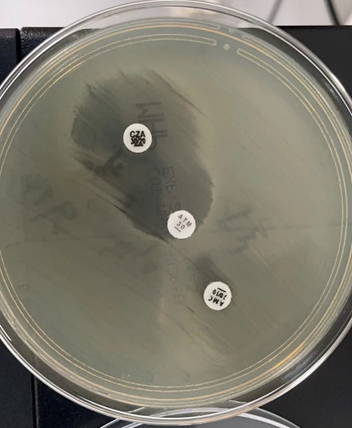


**Supplementary figure 2.** Combined susceptibility test of *S. maltophilia* by Kirby-Bauer disk diffusion. Aztreonam and ceftazidime-avibactam have synergistic effects

**Supplementary table 1.** Cytokine levels in blister fluid and blood samples

| Origin | Concentration (pg/ml) | | | | | | |
| --- | --- | --- | --- | --- | --- | --- | --- |
|  | IL-2 | IL-4 | IL-6 | IL-10 | TNF-α | IFN-γ | IL-17A |
| Blister fluid | 3.17 | 4.03 | 2265.91 | 96.67 | 2.91 | 1045.01 | 5.84 |
| Blood samples | 0.92 | 1.41 | 6.83 | 10.49 | 1.03 | 10.96 | 1.12 |

**Supplementary table 2.** Number of viable cells after 5 days

|  | Sample | | | | | |
| --- | --- | --- | --- | --- | --- | --- |
|  | T1 | T2 | T3 | C1 | C2 | C3 |
| Cell Concentration (cells/mL) | 1.56  x10^6^ | 1.62 x10^6^ | 1.48 x10^6^ | 1.56 x10^6^ | 1.60 x10^6^ | 0.81 x10^6^ |

**Supplementary table 3.** Cytokine concentrations in supernatants

| Sample | Concentration (pg/ml) | | | | | | |
| --- | --- | --- | --- | --- | --- | --- | --- |
|  | IL-2 | IL-4 | IL-6 | IL-10 | TNF-α | IFN-γ | IL-17A |
| T1 | 1.65 | 3.06 | 5.09 | 1.42 | 1.73 | 1.98 | 1.66 |
| T2 | 0.68 | 0.52 | 7.71 | 4.28 | 2.81 | 2.20 | 1.89 |
| T3 | 1.57 | 0.44 | 4.87 | 5.10 | 2.33 | 1.38 | 0.57 |
| C1 | 0.54 | 0.01 | 3.58 | 2.72 | 1.80 | 1.61 | 0.72 |
| C2 | 1.71 | 0.12 | 3.38 | 3.23 | 1.52 | 1.43 | 1.55 |
| C3 | 3.86 | 2.57 | 11369.81 | 257.72 | 117.18 | 137.90 | 409.90 |

Note:T1 is the test well for trimethoprim-sulfamethoxazole (TMP-SMX) at a final concentration of 21 μg/mL; T2 TMP-SMX test at 105 μg/mL; T3 TMP-SMX test at 210 μg/mL; C1 is the test well with DMSO;C2 is test well with media ; C3 is test well with PHA.

**Supplementary table 4.** Susceptibility results and interpretation for *S. maltophilia**

| Antimicrobial anent | MICs  (ug/ml) | Disk cotent  (ug) | Zone diameter  (mm) | Susceptibility |
| --- | --- | --- | --- | --- |
| Levofloxacin | ≥8 |  |  | R |
| Trimethoprim-sulfamethoxazole | ≥16/304 |  |  | R |
| Minocycline |  | 30 | 26 | S |
| Aztreonam |  | 30 | 6 | R |
| ceftazidime-avibactam |  | 30/20 | 29 | S |

Note: *: broth microdilution and Kirby-Bauer disk diffusion;Drug sensitivity was interpreted according to CLSI guidelines, due to the absence of breakpoints, *Pseudomonas spp.* breakpoints were used to categorize S.maltophilia; S, susceptible; R, resistant.

**Supplementary table 5.** Minimum inhibitory concentrations (MICs,µg/ml) of antifungal agents against the *Candida parapsilosis* 、*Fusarium solani* species complex and *Trichosporonas asahii*

| Strains | Antifungal MICs(ug/ml) | | | | | | | |
| --- | --- | --- | --- | --- | --- | --- | --- | --- |
|  | 5-FC | AMB | FCA | ITR | VRC | MCF | CAS | POC |
| *Candida parapsilosis* | <=1 | 1 | <=0.5 | NA | <=0.12 | 0.5 | 0.5 | NA |
| *Fusarium solani* species complex | NA | 2 | NA | 2 | 1 | NA | NA | 0.25 |
| *Trichosporonas asahii* | <=4 | <=0.5 | 2 | 0.125 | 0.125 | NA | NA | NA |

Note:5-FC,5-fluorocytosine; AMB,amphotericin B; FCA, fluconazole; ITR, itraconazole; VRC,voriconazole; MCF,micafungin; CAS, caspofungin; POC, posaconazole;NA:not available.

**Supplementary table 6.** Percentage of Treg and its subsets following the treatment

|  | Day 50 (%) | Day 60 (%) | Day 90 (%) |
| --- | --- | --- | --- |
| CD4^+^CD25^+^CD127low/CD4 | 6.2 | 8.2 | 14.1 |
| CD4^+^CD25^+^CD127lowCD45RA^+^/Treg | 41.9 | 6.4 | 3.9 |
| CD4^+^CD25^+^CD127lowCD45RA^-^/Treg | 58.1 | 93.6 | 96.1 |

Note:The expression of Treg and its subsets after the aGVHD accrued, Treg were gated on CD4^+^CD25^+^CD127low, and the subsets of Treg were divided into induced Treg (CD45RA^-^Treg) and naive Treg (CD45RA^+^Treg) and showed followed the time points after aGVHD accrued.
